# Reduction of Prep1 Levels Affects Differentiation of Normal and Malignant B Cells and Accelerates Myc Driven Lymphomagenesis

**DOI:** 10.1371/journal.pone.0048353

**Published:** 2012-10-25

**Authors:** Giorgio Iotti, Stefania Mejetta, Livia Modica, Dmitry Penkov, Maurilio Ponzoni, Francesco Blasi

**Affiliations:** 1 Laboratory of Transcriptional Regulation in Development and Cancer, IFOM (Fondazione Istituto FIRC di Oncologia Molecolare), Milano, Italy; 2 Department of Basic Medicine, Moscow State University, Moscow, Russia; 3 Department of Pathology, San Raffaele Scientific Institute, Milan, Italy; Case Western Reserve University, United States of America

## Abstract

The Prep1 homeodomain transcription factor has recently been recognized as a tumor suppressor. Among other features, haploinsufficiency of *Prep1* is able to strongly accelerate the B-lymphomagenesis in *E*μ*Myc* mice. Now we report that this occurs concomitantly with a change in the type of B-cell lymphomas generated by the *Myc* oncogene. Indeed, the tumors generated in the *E*μ*Myc-Prep1^+/−^* mice are much more immature, being mostly made up of Pro-B or Pre-B cells, while those in the *E*μ*Myc-Prep1^+/+^* mice are more differentiated being invariably IgM^+^. Moreover, we show that Prep1 is in fact required for the differentiation of Pro-B and Pre-B cells into IgM^+^ lymphocytes and/or their proliferation, thus showing also how a normal function of *Prep1* affects *E*μ*Myc* lymphomagenesis. Finally, we show that the haploinsufficiency of Prep1 is accompanied with a major decrease of Myc-induced apoptosis and that the haploinsufficieny is sufficient for all these effects because the second allele of *Prep1* is not lost even at late stages. Therefore, the tumor-suppressive activity of Prep1 is intertwined with both the interference with Myc-induced apoptosis as well as with natural developmental functions of the protein.

## Introduction

Expression of *Myc* in mouse B lymphocytes (*E*μ*Myc*) induces rapidly developing and highly penetrant B-cell lymphomas [1]. B cell progenitors, before the acquisition of functional surface IgM expression, are susceptible to transformation [2]. The latency and the rate of development of the tumors depends on the presence of active tumor suppressor functions, like p53 [3], Arf [4], Tip60 [5]. Moreover, it has been demonstrated that the survival of the mice relates to the stage of development of malignant cells [6].

The tumor suppressor *Prep1* [7] is a homeodomain transcription factor essential during early development [8]. The hypomorphic *Prep1^i/i^* mouse mutant expresses 3–10% of the protein and shows a leaky phenotype, lethal at E17.5 in 70% of the homozygous embryos, which is due to hematopoietic anomalies in all lineages [9]. The *Prep1^i/i^* embryos that escape embryonic lethality live an almost normal length life but a large percentage of them develops a variety of tumors, mainly lymphomas, within the first 18 months [7]. The null *Prep1* mutation in the heterozygous state (*Prep1^+/−^*), furthermore, drastically accelerates the development of *E*μ*Myc* tumors reducing their survival by at least half [7].

One of the main features of the *Prep1* deficient cells is the rapid accumulation of DNA damage, which we hypothesize favors the insurgence of mutations and hence malignancies [10]. However, the acceleration of lymphoma development in *Prep1^+/−^* mice might also be due to its role in the development of the B cell lineage. Indeed, we previously showed that *Prep1* is expressed in fetal liver B cell precursors and that its expression is critical in the early stages of B cell development [11].

In this paper we first show that *Prep1* is required for B cells development and maturation also in the adult mice and reiterate the effect of *Prep1* haploinsufficiency on the survival of the *E*μ*Myc* mice presenting a definitive survival curve. Moreover, we show that a large percentage of the tumors is enriched in less differentiated cells that are more resistant to Myc-induced apoptosis in the *Prep1^+/−^* background.

## Results

### Prep1 expression is necessary at the early stages of B cell development in adult mice

**Figure 1:**
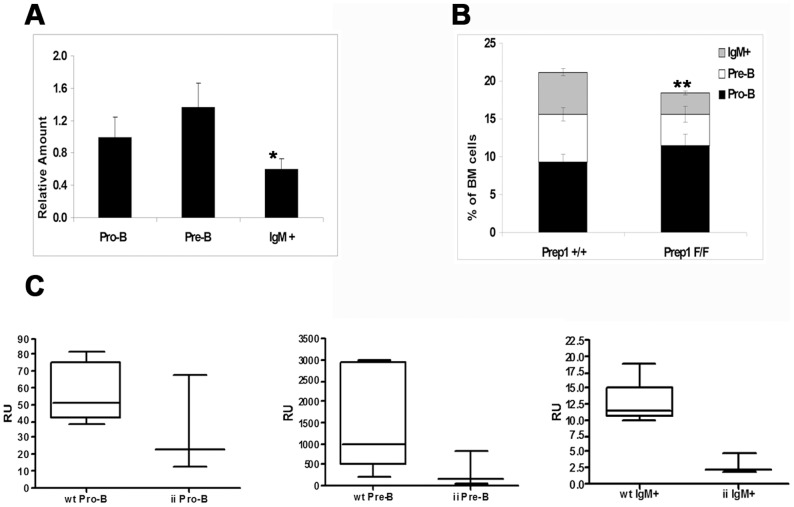
Role of Prep1 in B-cell development. A) Levels of Prep1 mRNA expression in BM B cell progenitors. Levels were normalized to the Pro-B group. Results were obtained from 3 independent 2-month old mice. * p<0.001. B) Effect of Prep1 deletion in B cell development. The plot represents the proportion of the different subtypes of B cell progenitors in BM of Rosa26Cre-ERT2 Prep1+/+ (n = 4) or Prep1F/F (n = 3) mice treated with tamoxifen (9 doses, 1 mg each). Pro-B, Pre-B and IgM+ populations are shown. p<0.01. C) Competitive repopulation of different B cell compartments. The repopulation activity was calculated as Repopulating Units (RU). In the box and whisker plots, RU of the different B cell subpopulations analyzed (Pro-B, Pre-B and IgM+) are shown. Data refer to 5 mice transplanted with 3 independent wt FL preparations and 3 mice transplanted with 2 Prep1^i/i^ FL preparations. Statistical significance of the differences between wt and Prep1^i/i^ results was evaluated with a Mann-Whitney test. p-values obtained were 0.25 for the Pro-B comparison, 0.071 for the Pre-B and 0.036 for the IgM+ compartment. The experiment was repeated twice with comparable results.

To study the expression of *Prep1* in adult B lymphopoiesis, we have sorted Pro-B (B220+/CD43+/CD25-/IgM−), Pre-B (B220+/CD43-/CD25+/IgM−) and more differentiated B (B220+/IgM+) cells from the bone marrow (BM) of two months old mice and measured Prep1 mRNA by Real Time PCR. As shown in **Fig. 1A**, Prep1 is expressed in the Pro-B and Pre-B cell fractions, but the levels decrease to approximately 50% in more differentiated cell populations (p<0.001). No statistically significant differences were detected between Pro-B and Pre-B subpopulations.

To examine Prep1 role in early B cell development in adult mice, we used an inducible Prep1 knock out system (**Fig. S1**). Either wt or *Prep1^F/F^* animals (see Methods) carrying the tamoxifen inducible Rosa26-CreERT2 transgene were intraperitoneally treated with tamoxifen (9 injections every other day, 1 mg/dose). Mice were sacrificed 11 days after the last injection and BM cells analyzed by FACS for Pro-B, Pre-B and Immature B cell populations. **Fig. 1B** shows that, upon deletion of Prep1, the Pro-B cell compartment expanded (11.5%±1% vs. 9.3%±1.5%), while the Pre-B (4.1%±1% vs. 6.3%±0.9%) and, more significatively, the IgM+ compartments (2.8%±0.3% vs. 5.5%±0.5%, p<0.01) were reduced.

The expansion of the pro-B cell compartment is cell-autonomous, as demonstrated by competitive repopulation experiments performed transplanting wt or *Prep1^i/i^* fetal liver (FL) cells into wild type lethally irradiated adult recipients (at a 1∶1 ratio) and analyzing splenic B cell subpopulations by flow cytometry in the BM two months after transplantation. The data are summarized in **Table S1**. In these experiments donor FL cells were distinguishable (CD45.2^+^) from the competitor wt BM cells (CD45.1^+^). We directly assessed the contribution of *Prep1^i/i^* cells to the different populations of B cell progenitors by measuring the repopulating units (RU, ratio between the percentage of donor CD45.2^+^ and competitor CD45.1^+^ cells) in the different subsets of B cell progenitors. As shown in **Fig. 1C**, while the repopulating activity of *Prep1^i/i^* cells is about 2 fold reduced in the Pro-B stage, in the more differentiated ones (Pre-B and IgM+) the difference increases to about 4 folds, suggesting that *Prep1* plays a role in the Pro-B to Pre-B cell transition. **Fig. S2** shows representative FACS analyses of BM CD45.2^+^ B220^+^ IgM^−^ cells stained with anti-CD43 and anti-CD25 antibodies from a mouse transplanted with wt FL cells and a mouse transplanted with *Prep1^i/i^* FL cells. Differences in the Pro-B and Pre-B cell populations is clearly appreciable. No differences were, on the other hand, detected within the competitor-derived (CD45.1^+^) cells. Statistical analysis with the Mann-Whitney test shows that the difference in progenitors is not statistically significant but gives a clear indication of trend. On the other hand, the reduction in differentiated IgM^+^ cells in *Prep1^i/i^* mice is statistically significant.

Therefore, these data, while confirming the previously published data showing that *Prep1^i/i^* FL cells compete less efficiently than wt [11], demonstrate also a direct role of Prep1 during the development of B cells probably at the Pro-B/Pre-B to IgM^+^ stage with a clear effect on the production of differentiated IgM^+^ cells. We conclude that Prep1 activity is required in the early stages of the physiological development of B cells.

### Prep1 haploinsufficiency accelerates the onset of myc-driven lymphomas by stimulating the insurgence of less differentiated tumors

**Figure 2:**
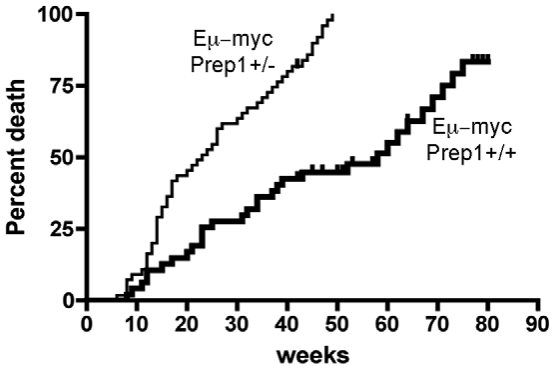
Prep1 haploinsufficiency in *E*μ*Myc* lymphomagenesis. Death rate curves for *E*μ*Myc* C57BL/6 transgenic mice carrying two wild-type (heavy line, n = 47) or one wild type and one deleted (fine line, n = 55) allele for *Prep1*. Median survival: 23 weeks for *Prep1^+/−^ E*μ*Myc*, 58 weeks for *E*μ*Myc* mice (*p*<0.001).

In order to assess whether alterations in Prep1 levels have a role in B cell malignancies, we crossed mice bearing two wt alleles (Prep1^+/+^) or one wt and one null allele for Prep1 (*Prep1^+/−^*) with mice constitutively expressing c-Myc under the control of the enhancer of the heavy chain of immunoglobulins (*E*μ*Myc*). As previously reported [7], an accelerated onset and enhanced penetrance of Myc-induced B-cell lymphomas was observed in the *Prep1^+/−^* background. **Fig. 2** shows an updated (with respect to the one published in ref. [7]) survival curve in which the number of mice is higher and the observation period increased. The median survival time in the *Prep1* wild type background is 58 weeks, compared to the 23 weeks (p<0.001) in the *Prep1^+/−^*background.

In order to characterize lymphomas arisen in the two cohorts, we performed extensive immunophenotyping of 11 *E*μ*Myc*Prep1^+/+^ and 22 *E*μ*Myc* Prep1^+/−^ mice using antibodies for markers characterising different subsets of early B cells: anti-B220, anti-IgM and anti-CD43. Lymphomas were classified as Pro-B (B220+/IgM−/CD43+), Pre-B (B220+/IgM−/CD43-) or B (B220+/IgM+) based on the predominant spleen population detected by FACS. **Table 1** shows the proportion of Pro-B, Pre-B and B lymphomas in the two groups. In particular, Pro-B lymphomas were exclusively observed in the *E*μ*Myc*Prep1^+/−^ mice, in the proportion of 45%. While the vast majority (81.8%) of *E*μ*Myc*Prep1^+/+^ mice developed B-lymphomas, the *E*μ*Myc*Prep1^+/−^ mice developed more immature lymphomas (54.5%) with a clear predominance of tumours derived from Pro-B cells (p<0.05, proportion of B-lymphomas in *E*μ*Myc*Prep1^+/+^ vs. *E*μ*Myc*Prep1^+/−^ mice, Fisher's exact test). All analyzed spleens stained negatively for early progenitor markers (AA4.1 and c-kit). The B220^+^/IgM^+^ malignant spleens shows immunophenotypic markers characteristic of immature B cells, staining negatively for both CD21 and CD23. In **Fig. S3A**, we report examples of lymphomas developed in *E*μ*MycPrep1*+/+ and *E*μ*MycPrep1*+/− mice, respectively. Reduced surface cytofluorimetric IgM staining in *E*μ*MycPrep1*+/− lymphomas always correlated with a strong reduction in the expression of IgM by intracellular immunofluorescence (**Fig. S3B**) and reduced levels of expression of the heavy chain (µ-chain) by immunoblotting (**Fig. S3C**).

**Table 1:** Immunophenotyping of the *E*μ*Myc* lymphomas in Prep1^+/+^ v. Prep+/−^+^mice.

Immunophenotype	*E*μ*Myc -*Prep1^+/+^	*E*μ*Myc -*Prep1^+/−^	*p-value* *
Pro-B (B220^+^, IgM^−^, CD43^+^)	0	10 (45.5%)	0.013
Pre-B (B220^+^, IgM^−^, CD43^−^)	2 (18.2%)	2 (9%)	1
B (B220+, IgM^+^)	9 (81.8%)	10 (45.5%)	0.07

Tumoral splenic cells from eleven *E*μ*Myc-Prep1^+/+^* and twentytwo *E*μ*Myc-Prep1^+/−^* mice have been analyzed by flow cytometry for the markers indicated.

* Fisher's exact test.

However, the survival of mice in the indicated groups was not correlated with the immunophenotyping. Indeed the median survival of *E*μ*Myc*Prep1+/− mice was 19 weeks independently of the type of tumour developed (**Fig. S3D**).

**Figure S3E** shows an example of a hematoxylin-eosin staining of a lymphnode from a tumor that flow-cytometricallywas considered pro-B (IgM_−_, CD43^+^).

These results clearly indicate that reduction of Prep1 levels favours development of lymphomas dominated by undifferentiated progenitors. However, possibly due to the limited sample size, we could not find a correlation between survival and tumor differentiation level unlike what previously reported [6].

### Prep1^+/−^ *E*μ*Myc* tumors don't lose the wt Prep1 allele

Lymphomas developed in heterozygous mice did not lose the wildtype *Prep1* allele, revealing that there is not a selective pressure for the complete inactivation of the *Prep1* locus (**Figure S4A**). Prep1 is still expressed at the protein level in all tumors with no difference in its subcellular localization (**Figure S4B**). Furthermore, sequencing analysis of several *E*μ*MycPrep1*+/+ and *E*μ*MycPrep1*+/− tumors never showed any mutation in the coding sequence of the *Prep1* mRNA (not shown). The absence of loss of heterozygosity indicates that *Prep1* acts as a haploinsufficient tumour suppressor at early stages of lymphomagenesis.

### *Prep1* ^+/−^ B cell progenitors proliferate more and are more resistant to myc-induced apoptosis

**Figure 3:**
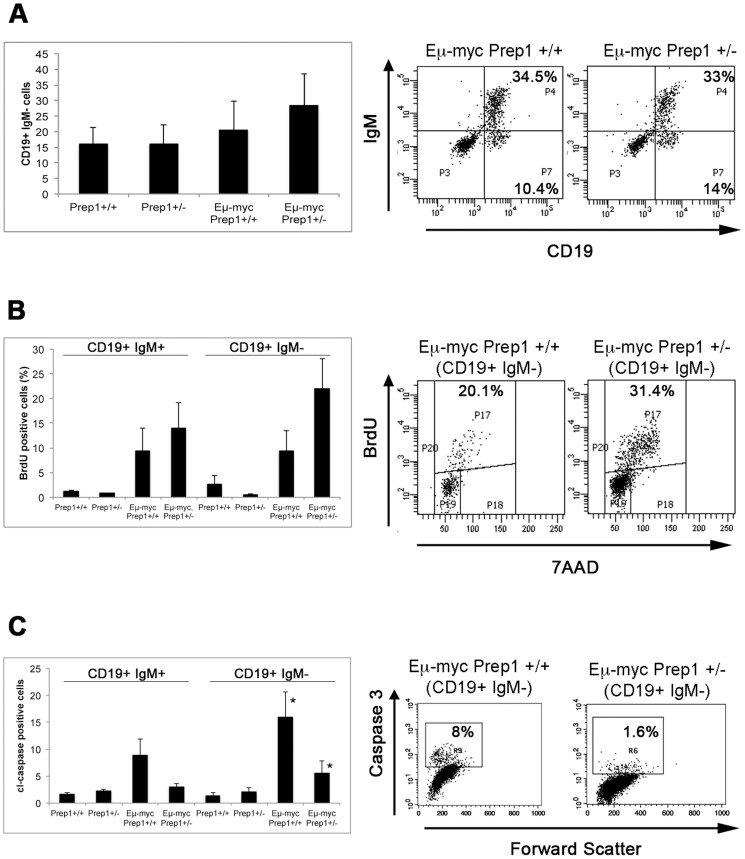
B cell analysis in pre-tumoral mice. A) Splenic cells of the indicated groups were stained with antibodies recognizing CD19 and IgM. Proportion of CD19+ IgM− cells is reported. B) Mice of the indicated groups were injected intraperitoneally with BrdU 2 hours before their sacrifice. Splenic cells were incubated with antibodies against CD19, IgM and BrdU. The plot indicates proportion of BrdU positive cells in the CD19+/IgM− subpopulation. Representative dot plots of the CD19^+^ IgM^−^ cells are on the right. C) Splenic cells were incubated with antibodies against CD19, IgM and cleaved Caspase 3. Percentage of cl-Casp3 positive cells in the CD19^+^/IgM^+^ and in the CD19^+^/IgM^−^ population is reported (* p<0.05 *E*μ*Myc Prep1^+/+^* CD19^+^/IgM^−^ vs. *E*μ*Myc Prep1* ^+/−^ CD19^+^/IgM^−^). Representative dot plots of the CD19+/IgM^−^-gated cells are on the right.

We further explored how *Prep1* heterozygosity affects the biology of Myc-induced lymphomagenesis by analyzing mice of the four genotypes (*Prep1*+/+, *Prep1*+/−, *E*μ*MycPrep1*+/+, *E*μ*MycPrep1*+/−) at a pre-tumoral stage (2-months old). We analyzed 3 *Prep1*+/+, 3 *Prep1*+/−, 6 *E*μ*MycPrep1*+/+ and 7 *E*μ*MycPrep1*+/− mice from 3 independent litters. The proportion of the different B cell populations in the spleen of the analyzed mice showed a minor, and not statistically significant, increase in the percentage of B cell progenitors (CD19+ IgM−) in the *E*μ*MycPrep1*+/− group (**Fig. 3A**).

Interestingly, we found an increased proportion of proliferating and a decreased proportion of apoptotic splenic B cell in the *E*μ*MycPrep1*+/− group, if compared to the wt counterpart. The effect was much more marked in the more undifferentiated (CD19^+^ IgM^−^) B cell progenitor compartment, in which about twice as many cells in the *E*μ*MycPrep1*+/− group were proliferating in a BrdU labelling assay (21.2% vs. 10.2%, p = 0.18, **Fig. 3B**) and a lower proportion of the same population of cells was positive for cleaved caspase 3 (5.6% vs. 15.9%, p<0.05, **Fig. 3C**). However, the difference in apoptosis is not specific to progenitor cells being present also in the more differentiated Igm^+^ compartment (**Fig. 3C**). **Figures 3B and 3C** show representative flow ytometry plots. The result of these experiments indicates that myc-induced apoptosis is strongly counter-balanced under conditions of *Prep1* haploinsufficiency.

Thus, the *Prep1*-dependent increased proliferative and the decreased apoptotic activities indicate a possible mechanism favouring lymphomagenesis.

## Discussion

Homeodomain transcription factors are essential in patterning of all mammalian tissues during embryonic development and adult life homeostasis [12]. Furthermore, they play a pivotal role in the maintenance and functionality of stem cells. In particular, it has been demonstrated that members of the TALE (three-aminoacid loop extension) class of transcription factors are critical in the development of virtually all embryonic and adult hematopoietic lineages. Pbx1−/− embryos exhibit profound anemia and decrease in common myeloid progenitor cells in the fetal liver [13]; Pbx1 is also essential for generation of common lymphoid progenitors [14] and maintenance of LT-HSC [15]. Meis1-deficient embryos lack megakaryocytes and LT-HSC [16], [17]. Prep1 is involved in T cell lymphopoiesis in the adult [18] and in general hematopoiesis in the embryo [9] and is required for the maintenance of long-term repopulating hematopoietic stem cells [11].

In the analysis of the present data, it must be born in mind that apparently minor differences in the level of Prep1 may have important physiological effects. An example of this is the phenotype of Prep1-deficient mice. The Prep1 “null” (*Prep1^−/−^)* embryo, that expresses no Prep1, dies at E7.5 [10]. The hypomorphic *Prep1^i/i^* embryos, that express 2% of the normal Prep1 mRNA, mostly die at E17.5 or later [9]. A double heterozygous mouse (*Prep1^−/i^*), instead, dies at E12.5 [23]. Hence the difference between 1 and 2% Prep1 mRNA has a strong impact on mouse development.

*E*μ*-myc* is a tumor model widely used to explore the function of putative oncogenes and tumor suppressor genes [3], [19], [20]. We have demonstrated that the homeodomain transcription factor Prep1 is a tumor suppressor: indeed, the homozygous *Prep1^i/i^* hypomorphic mice that survive embryonic lethality are prone to develop tumors and a survey of human cancers shows a dramatic reduction of *Prep1* expression in a large proportion (70%) of the patients. Consistently, *Prep1* haploinsufficiency strongly accelerates *E*μ*-myc* lymphomagenesis [7]. In fact, we recently demonstrated that the tumor suppressor function of Prep1 is associated to its role in the maintenance of genomic stability [10]. The Prep1 function in myc-driven tumorigenesis is, nevertheless, still unclear.

Here we show that in *Prep1*-haploinsufficient mice the acceleration of myc-induced lymphomagenesis correlates with the lower degree of differentiation of the tumors and with the increased proliferation rate and resistance to Myc-induced apoptosis of the pre-tumoral B cell progenitors. In the bone marrow, Prep1 mRNA levels are higher in undifferentiated progenitors (Pro-B and Pre-B) compared to mature B cells (**Fig. 1A**). Moreover, Prep1 is necessary for the correct Pro-B to immature B cell transition (**Fig 1B and 1C**), as this ratio is increased in the Prep1 Ko background. Since the analysis of B cells development was not carried out in a murine model of complete lack of *Prep1* expression, residual Prep1 levels probably prevent the observation of a stronger B cell phenotype. Indeed, the efficiency of deletion in the Prep1 conditional Ko was at best 70–80% (**Fig. S1B**).

The role of *Prep1* in B cells development appears to be somewhat different from *Pbx1*, since the latter is indispensable earlier in the developmental process, for the generation of the common lymphoid progenitors (14), while Prep1-deficient animals show a relative accumulation of Pro-B cells and a dramatic reduction of immature cells (half of wild type upon inducible deletion of *Prep1*, **Fig. 1B**). However, it is not impossible that this apparent discrepancy between *Pbx1* and *Prep1* is due to the inefficient deletion in the conditional Ko and the residual expression of the *Prep1* gene in the hypomorphic mice.

During B-lymphocytes maturation, Prep1 might either directly affect the differentiation genetic machinery or indirectly affect a basic property of the cells (like proliferation or survival) leading to an apparent developmental block. Although present data do not address this question, we notice that the BrdU incorporation of *Prep1^+/−^* heterozygous progenitors is decreased whereas no difference is observed in the IgM^+^ compartment (**Fig. 3B**). On the other hand, no real difference in apoptosis is evident (**Fig. 3C**). Therefore, the effect of Prep1 reduction might be at the level of proliferation rather than differentiation.

The detailed analysis of the process of *E*μ*-myc* lymphomagenesis in *Prep1* wt and heterozygous mice demonstrated that Prep1 functions as a haploinsufficient tumor suppressor at the pre- or early stages of tumor development, since neither loss of heterozygosity nor mutations were observed in the *Prep1* locus (**Fig S3**). In *E*μ*-mycPrep1* ^+/−^ mice there was a clear increase in Pro-B or Pre-B lymphomas compared to *E*μ*-mycPrep1* ^+/+^ (**Table 1**). This indicates that the reduction of Prep1 favours lymphomas dominated by undifferentiated progenitors. Even though, possibly due to the limited sample size, we could not find a correlation between survival and tumor differentiation level as reported by others [6], it is still possible that *Prep1*-dependent defects in B-cell development explain the acceleration of lymphoma onset. However, a more plausible explanation is given by the significant reduction of myc-driven apoptosis and by the increased proliferation of *E*μ*-mycPrep1*+/− B cell progenitors (**Fig 3C**). Thus, wild type levels of Prep1 are necessary to elicit the full pro-apoptotic effect of *Myc* over-expression in these cells. It will be important to identify the molecular basis for the difference between *E*μ*-mycPrep1*+/− and *E*μ*-mycPrep1*+/− B cell progenitors to identify the Prep1-dependent mechanism allowing oncogene-induced apoptosis and progress towards the full blown lymphoma.

In general, genetic deficiencies leading to arrested B cell development and accumulation of early B cell precursors significantly enhance lymphoma development in *E*μ*-myc* mice. It has recently been demonstrated that the mismatch repair (MMR) pathway suppresses mutations complementing c-Myc-associated oncogenesis during early B cell development [21]. It is possible that, consistently with its role in maintenance of genomic stability [10], the presence of Prep1 actively suppresses the acquisition of secondary mutations that coordinate with c-Myc to transform B cells.

## Materials and Methods

### Mice

*Prep1^i/i^* and *E*μ*Myc* Prep1^+/−^ mice have been described [9], [10].

Prep1 floxed mice (C57BL/6 background) possess loxP sites flanking Prep1 exons 6 and 7 that can be deleted after Cre mediated recombination. These mice were crossed with Rosa26CREERT2 mice (ERT2) [22], [23] to generate ERT2+ Prep1F/F mice and ERT2+ Prep1+/+ control mice. Genotypes were determined by genomic PCR on DNA preparations from tail biopsies using the following primers: Prep1 upsteam loxP site RV 5′ATTGATGGTGCCAACCAAGTG3′, FW 5′GACTAAAGGTACAGATAAGGGC3′; Prep1 downsteam loxP site RV 5′GGCACATCGTGAAGTTGGG3′ FW 5′GCAGGTTAGAAAGGGAGGAC3′; Rosa26CREERT2 FW 5′ ACGAACCAAGGTGACAGCAATG3′, RV 5′ CTCGACCAGTTTAGTTACCC3′. Cre-mediated deletion was induced in 8 weeks old mice by treating mice intraperitoneally with 9 injections of tamoxifen (Sigma) every other day (1 mg tamoxifen/injection). 11 days after the last injection mice were sacrified and bone marrow cells were analysed.

### Real Time PCR

Total RNA was extracted from bone marrow cells according to standard procedure using the Qiagen kit. cDNA was synthesized from 1 µg of total mRNA and reverse-transcription was performed (SuperScriptII Reverserse Transcriptase, Invitrogen) following the manufacturer's instructions. Real-time PCR (Sybr Green technology, Applied Biosystems) was employed to quantify the Cre-mediated deletion efficiency. Glyceraldehyde 3-phosphate dehydrogenase (Gapdh) mRNA was used as internal control for each sample and all reactions were run in triplicate. Primers to detect Prep1+/+ mRNA: FW 5′ TGAACCAAGACCTCAGCATCT 3′, RW 5′GCAGGACACCCCTCTTGTT 3′.

### Immunofluorescence, flow cytometry analysis and cell sorting

For splenic cell immunofluorescence, cells were cytospun onto slides and fixed with methanol/acetone (4∶1). Cells were stained with FITC-conjugated anti-IgM (Jackson ImmunoResearch Laboratories). Nuclei were counterstained with DAPI (Sigma).

Cell surface marker antibodies were purchased from eBioscience. FACS analysis was performed in a FACS Calibur and cells sorted using a FACS Aria (BD Biosciences).

### Competitive repopulation assay

Lethally irradiated CD45.1^+^ C57BL/6J mice were inoculated with 500000 donor (wt or *Prep1^i/i^*) CD45.2^+^ fetal liver cells together with 500000 competitor CD45.1^+^ wt bone marrow cells. 8 weeks after transplantation, mice were sacrificed and bone marrow repopulation analysed by flow cytometry.

### In vivo proliferation and apoptosis assays

For proliferation assays, mice were intraperitoneally injected with BrdU (1 mg) 2 hours before their sacrifice. Splenic cells were recovered and stained following manufacturer instructions (FITC BrdU Flow kit, BD Biosciences).

For apoptosis assays, an anti-cleaved caspase-3 antibody (Asp 175, Cell Signaling Technology) was used.

Stained cells were analysed by FACS.

### Loss of heterozygosity analysis

Genomic DNA was extracted with QIAGEN Genomic-tips (100/G) from tail or lymphnode biopsies. 100 ng per sample were used in PCR reactions. Primers designed for genotyping of the *E*μ*Myc* Prep1^+/−^ strain were used.

## Supporting Information

Figure S1 **A) Schematic model of the inducible ERT2+ Prep1F/F mouse model. B) Prep1 mRNA levels upon inducible gene knock out.** Real Time PCR analysis of bone marrow cells derived from ERT2+ Prep1+/+ and ERT2+ Prep1F/F mice after tamoxifen induction. Data are normalized to Prep1 levels in ERT2+ Prep1+/+ cells (n: 4 ERT2+ Prep1+/+ and 3 ERT2+ Prep1F/F).
(TIF)

Figure S2 **Representative FACS analysis of Pro-B and Pre-B compartments in donor-derived transplanted cells.** Bone marrow cells were initially gated for the CD45.2 v. CD45.1 marker (Top), then assayed for the B cells markers B220 and IgM (middle panels) and finally for their Pro-B v. Prep-B nature (bottom panel). All the plots on the left side refer to mice transplanted with wt FL cells while those on the right side refer to mice transplanted with *Prep1^i/i^* FL cells.
(TIF)

Figure S3 **Characterization of the Prep1+/− lymphomas.** A) Representative FACS analysis of EμMyc *Prep1^+/+^* and EμMyc *Prep1^+/−^* lymphomas. Splenic cells were stained with anti-B220 and anti-IgM. The *Prep1^+/+^* tumor is largely enriched of B220^+^/IgM^+^ cells, the *Prep1^+/−^* one is composed of B220^+^/IgM^−^ cells. B) Representative immunofluorescent staining of *EμMyc Prep1^+/+^* and *EμMyc Prep1^+/−^* lymphomas. Splenic cells were cytospun onto slides, fixed with methanol/acetone and stained with FITC-conjugated anti-IgM. Nuclei were counterstained with DAPI. C) Immunoblotting analysis of *EμMyc Prep1^+/+^* and *EμMyc Prep1^+/−^* lymphomas. Total lysates from splenocytes of two *E*μ*Myc Prep1^+/+^* and seven *E*μ*Myc Prep1^+/−^* lymphomas (three of which negatively staining for IgM by FACS) were analyzed by Western blotting using an antibody recognizing the heavy chain of immunoglobulins (μ-chain). Extracts of wt mouse embryonic fibroblasts (MEF) and normal spleen (SPL) were loaded as negative and positive control, respectively. Anti tubulin was used as loading control. D) Median survival of Pro-B, Pre-B and B cell lymphomas. The plot indicates median survival of mice affected by the indicated type of lymphomas belonging to the *E*μ*Myc Prep1^+/+^* or the *E*μ*Myc Prep1^+/−^* group. E) Hematoxylin-Eosin staining on one section of a Pro-B tumor in the *E*μ*Myc Prep1^+/−^* group**.**
(TIF)

Figure S4 ***EμMyc Prep1^+/−^*** **do not lose the normal Prep1 allele. A) Loss of heterozigosity in** ***E*** **μ** ***Myc*** **lymphomas.** PCR genotyping was performed in extracts from one *E*μ*Myc Prep1^+/+^* and three *E*μ*Myc Prep1^+/−^* mice. Normal (N) samples were derived from tail DNA obtained at the moment of mouse weaning, Tumor (T) samples were derived from lymphnodes at the moment of mouse sacrifice. **B) Levels and subcellular localization of Prep1 in normal and tumoral lymphnodes.** Immunoblotting of Prep1 was performed was performed in nuclear (N) and cytoplasmic (C) extracts obtained from wt thymus and lymphnodes and from lymphnode samples of two *E*μ*Myc Prep1^+/+^* lymphomas, two *E*μ*Myc Prep1^+/−^* lymphomas positive for IgM and two *E*μ*Myc Prep1^+/−^* lymphomas negative for IgM. Levels of vinculin and H3 are reported as marker of cytoplasmic and nuclear extracts, respectively.
(TIF)

Table S1 **Immunophenotyping of the** ***E*** **μ** ***Myc*** **lymphomas in** ***Prep1^+/+^*** **v.** ***Prep1^+/−^*** **mice.** Tumoral splenic cells from eleven *E*μ*Myc-Prep1^+/+^* and twentytwo *E*μ*Myc-Prep1^+/−^* mice have been analyzed by flow cytometry for the markers indicated. * Fisher's exact test.
(DOCX)
