# Supplementary figures and images for: Reduction of Prep1 Levels Affects Differentiation of Normal and Malignant B Cells and Accelerates Myc Driven Lymphomagenesis
Source: PLoS One. 2012 Oct 25;7(10):e48353. doi: 10.1371/journal.pone.0048353 (PMC3485025; doi:10.1371/journal.pone.0048353)

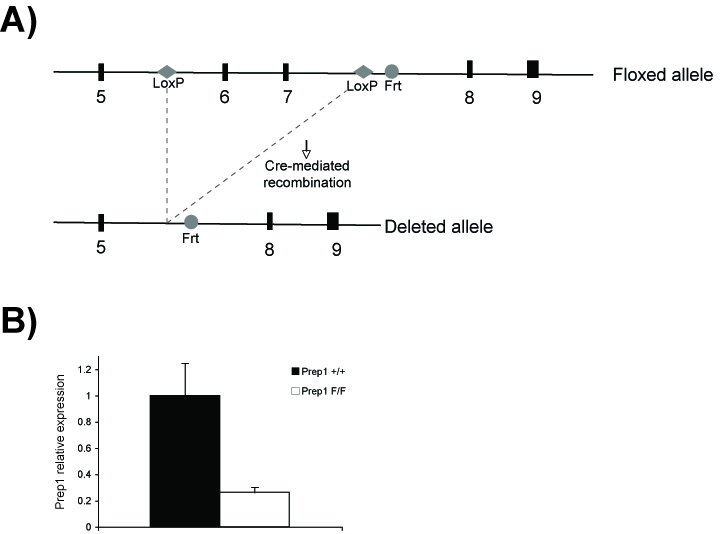

Supplement: Figure S1 — A) Schematic model of the inducible ERT2+ Prep1F/F mouse model. B) Prep1 mRNA levels upon inducible gene knock out. Real Time PCR analysis of bone marrow cells derived from ERT2+ Prep1+/+ and ERT2+ Prep1F/F mice after tamoxifen induction. Data are normalized to Prep1 levels in ERT2+ Prep1+/+ cells (n: 4 ERT2+ Prep1+/+ and 3 ERT2+ Prep1F/F). (TIF) [file pone.0048353.s001.tif]

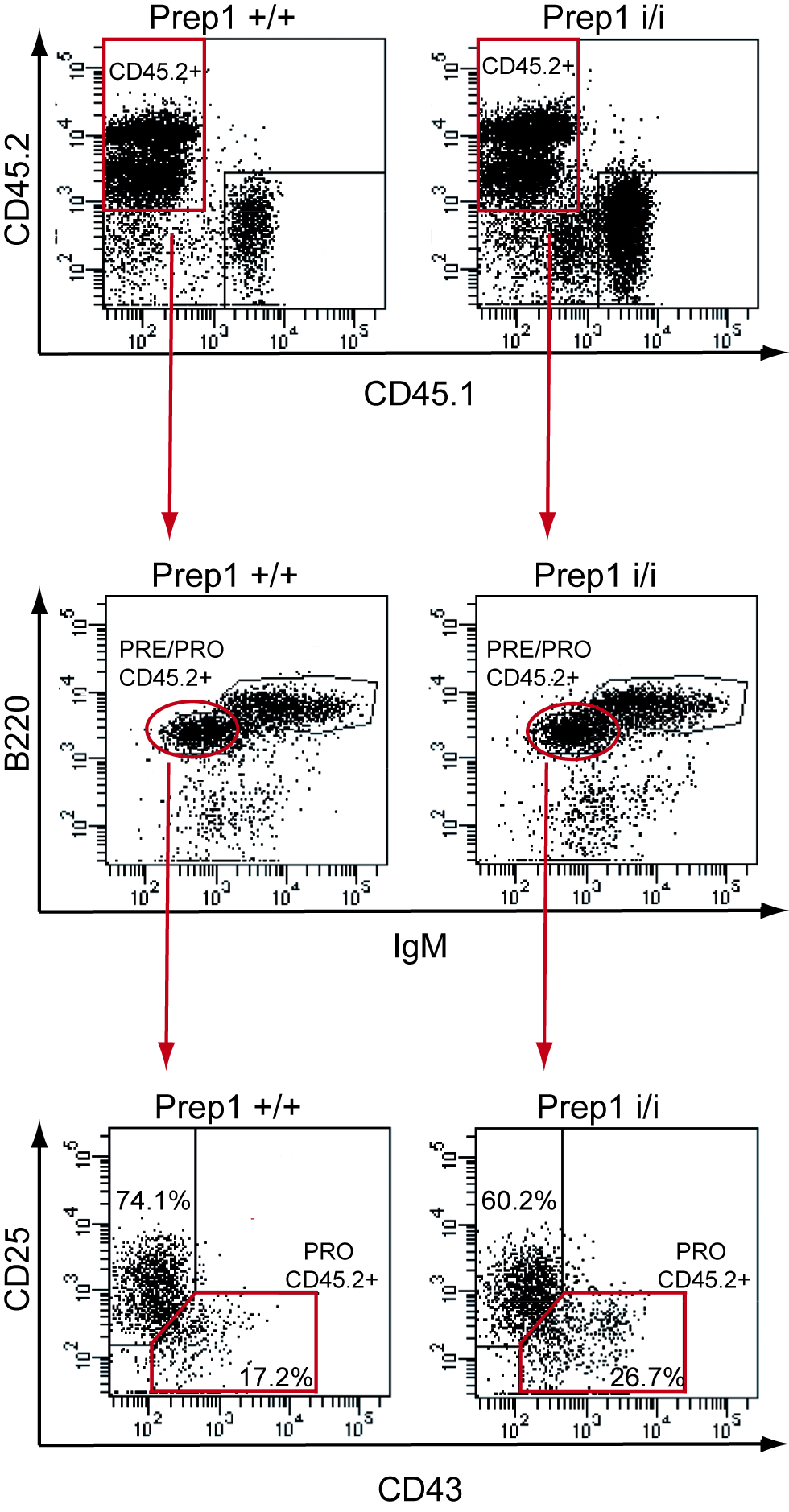

Supplement: Figure S2 — Representative FACS analysis of Pro-B and Pre-B compartments in donor-derived transplanted cells. Bone marrow cells were initially gated for the CD45.2 v. CD45.1 marker (Top), then assayed for the B cells markers B220 and IgM (middle panels) and finally for their Pro-B v. Prep-B nature (bottom panel). All the plots on the left side refer to mice transplanted with wt FL cells while those on the right side refer to mice transplanted with Prep1i/i FL cells. (TIF) [file pone.0048353.s002.tif]

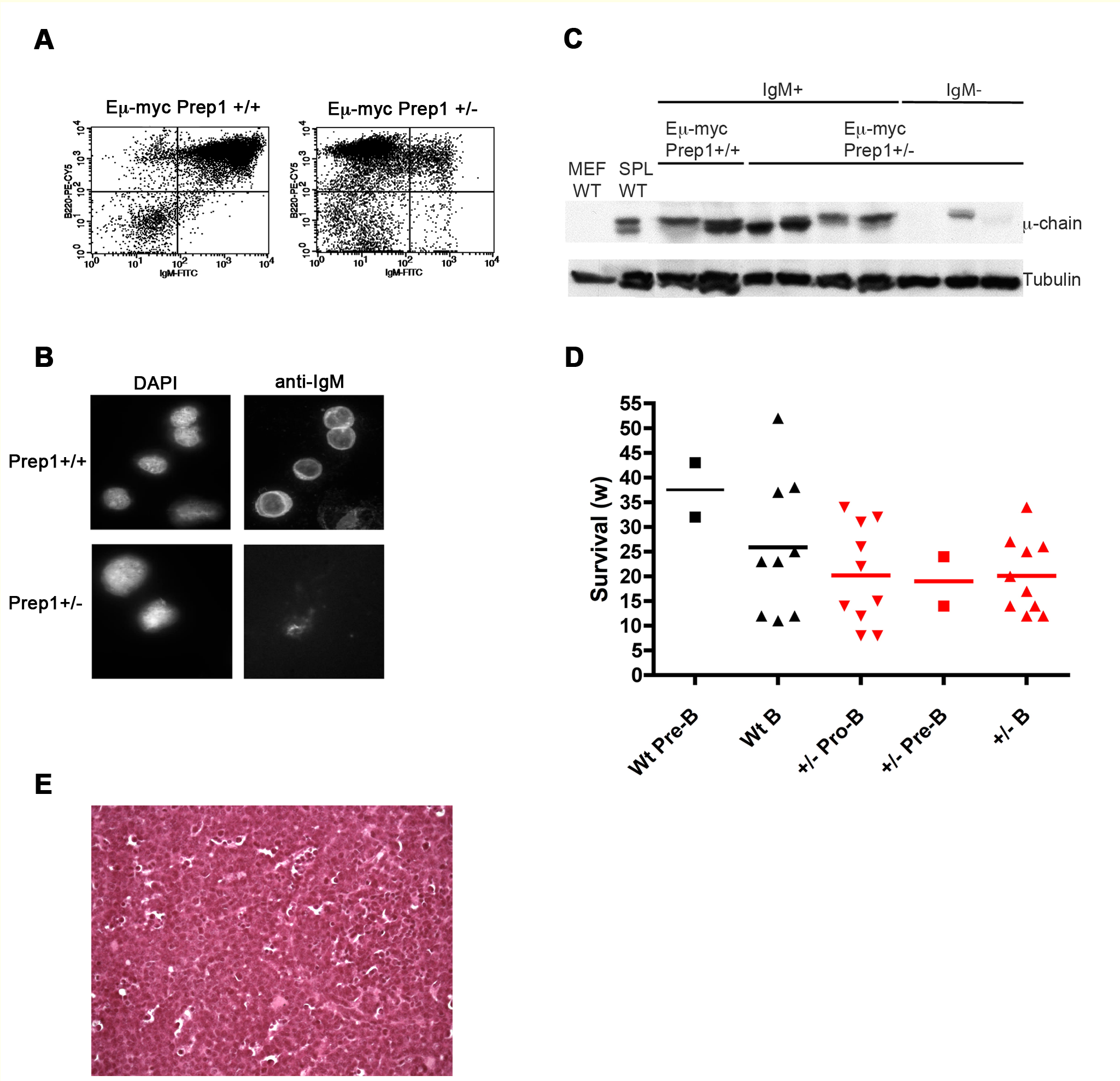

Supplement: Figure S3 — Characterization of the Prep1+/− lymphomas. A) Representative FACS analysis of EμMyc Prep1+/+ and EμMyc Prep1+/− lymphomas. Splenic cells were stained with anti-B220 and anti-IgM. The Prep1+/+ tumor is largely enriched of B220+/IgM+ cells, the Prep1+/− one is composed of B220+/IgM− cells. B) Representative immunofluorescent staining of EμMyc Prep1+/+ and EμMyc Prep1+/− lymphomas. Splenic cells were cytospun onto slides, fixed with methanol/acetone and stained with FITC-conjugated anti-IgM. Nuclei were counterstained with DAPI. C) Immunoblotting analysis of EμMyc Prep1+/+ and EμMyc Prep1+/− lymphomas. Total lysates from splenocytes of two EμMyc Prep1+/+ and seven EμMyc Prep1+/− lymphomas (three of which negatively staining for IgM by FACS) were analyzed by Western blotting using an antibody recognizing the heavy chain of immunoglobulins (μ-chain). Extracts of wt mouse embryonic fibroblasts (MEF) and normal spleen (SPL) were loaded as negative and positive control, respectively. Anti tubulin was used as loading control. D) Median survival of Pro-B, Pre-B and B cell lymphomas. The plot indicates median survival of mice affected by the indicated type of lymphomas belonging to the EμMyc Prep1+/+ or the EμMyc Prep1+/− group. E) Hematoxylin-Eosin staining on one section of a Pro-B tumor in the EμMyc Prep1+/− group. (TIF) [file pone.0048353.s003.tif]

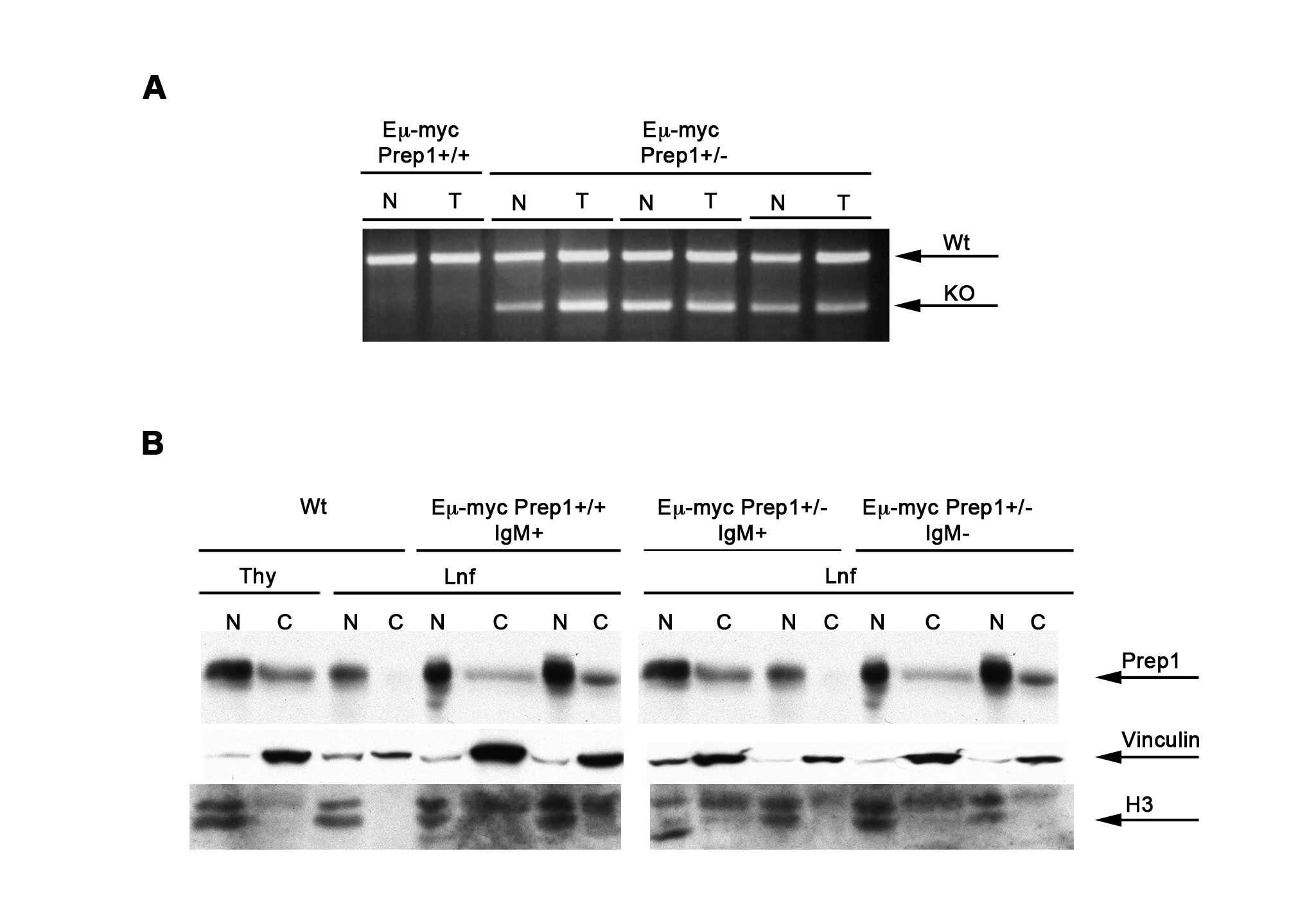

Supplement: Figure S4 — EμMyc Prep1+/− do not lose the normal Prep1 allele. A) Loss of heterozigosity in E μ Myc lymphomas. PCR genotyping was performed in extracts from one EμMyc Prep1+/+ and three EμMyc Prep1+/− mice. Normal (N) samples were derived from tail DNA obtained at the moment of mouse weaning, Tumor (T) samples were derived from lymphnodes at the moment of mouse sacrifice. B) Levels and subcellular localization of Prep1 in normal and tumoral lymphnodes. Immunoblotting of Prep1 was performed was performed in nuclear (N) and cytoplasmic (C) extracts obtained from wt thymus and lymphnodes and from lymphnode samples of two EμMyc Prep1+/+ lymphomas, two EμMyc Prep1+/− lymphomas positive for IgM and two EμMyc Prep1+/− lymphomas negative for IgM. Levels of vinculin and H3 are reported as marker of cytoplasmic and nuclear extracts, respectively. (TIF) [file pone.0048353.s004.tif]
