# Supplementary material for: Reduction of Prep1 Levels Affects Differentiation of Normal and Malignant B Cells and Accelerates Myc Driven Lymphomagenesis
Source: PLoS One. 2012 Oct 25;7(10):e48353. doi: 10.1371/journal.pone.0048353 (PMC3485025; doi:10.1371/journal.pone.0048353)
Supplement: Table S1 — Immunophenotyping of the E μ Myc lymphomas in Prep1+/+ v. Prep1+/− mice. Tumoral splenic cells from eleven EμMyc-Prep1+/+ and twentytwo EμMyc-Prep1+/− mice have been analyzed by flow cytometry for the markers indicated. * Fisher's exact test. (DOCX) [file pone.0048353.s005.docx]

**Table S1**

**Competitive repopulation experiments show a decreased repopulation of differentiated B cells in the Prep1^i/i^ mice*.**

| **Population analysed** | **Total BM** | | **Pro-B** | | **Pre-B** | | **IgM+** | |
| --- | --- | --- | --- | --- | --- | --- | --- | --- |
| **Genotype** | +/+ | i/i | +/+ | i/i | +/+ | i/i | +/+ | i/i |
| **Median°** | 89.37 | 73.09 | 98.09 | 95.81 | 99.90 | 99.36 | 91.99 | 68.47 |
| **Range** | 88.02-92.68 | 67.46-85.94 | 97.46-98.78 | 92.90-98.55 | 99.51-99.97 | 98.79-99.88 | 90.99-94.99 | 66.33-83.19 |

* The data represent the percentage of CD45.2^+^ cells of the experiment shown in Figure 1C.

° The table reports the percentage of CD45.2^+^ in the different BM celland populations. Median and range refer to 5 mice transplanted with wt FL and 3 mice transplanted with Prep1^i/i^ FL. The cyto-fluorimetric phenotype of Pro-B, Pre-B and IgM+ cells is described in the text.
